# Therapeutic Efficacy of Urethral Sphincteric Botulinum Toxin Injections for Female Sphincter Dysfunctions and a Search for Predictive Factors

**DOI:** 10.3390/toxins13060398

**Published:** 2021-06-02

**Authors:** Yin-Chien Ou, Kuan-Hsun Huang, Hau-Chern Jan, Hann-Chorng Kuo, Yao-Lin Kao, Kuen-Jer Tsai

**Affiliations:** 1 Institute of Clinical Medicine, College of Medicine, National Cheng Kung University, Tainan 704, Taiwan; i54921051@gmail.com; 2 Department of Urology, National Cheng Kung University Hospital, College of Medicine, National Cheng Kung University, Tainan 704, Taiwan; 3 Department of Urology, Dalin Tzu Chi Hospital, Buddhist Tzu Chi Medical Foundation, Chiayi 622, Taiwan; i5490108@hotmail.com; 4 Division of Urology, Department of Surgery, National Cheng Kung University Hospital Dou-Liou Branch, Yunlin 640, Taiwan; jan.hauchern@gmail.com; 5 Department of Urology, Hualien Tzu Chi Hospital, Buddhist Tzu Chi Medical Foundation and Tzu Chi University, Hualien 970, Taiwan; hck@tzuchi.com.tw; 6 Research Center of Clinical Medicine, National Cheng Kung University Hospital, College of Medicine, National Cheng Kung University, Tainan 704, Taiwan

**Keywords:** botulinum toxin, urethral sphincter, voiding dysfunction, female

## Abstract

External urethral sphincter (EUS) dysfunction is a common, bothersome female voiding dysfunction. This study aims to analyze the characteristics of different types of female EUS dysfunction, as well as to determine the outcome predictors of sphincteric botulinum toxin A (BoNT-A) injection. Women receiving sphincteric BoNT-A injections for refractory EUS dysfunction were retrospectively reviewed. A comparison of the baseline clinical, urodynamic parameters and the treatment responses were made for patients with different EUS dysfunctions. A total of 106 females were included. Significantly increased detrusor overactivity, detrusor contracting pressure and the bladder outlet obstruction index with decreased urge sensation were noted in patients diagnosed with dysfunctional voiding or detrusor sphincter dyssynergia comparing to those diagnosed with poor relaxation of the external urethral sphincter. The average subjective improvement rate was 67% for the injection. The therapeutic effect was not affected by the type of EUS dysfunction. The multivariate analysis revealed that bladder neck narrowing and catheterization history were predictive of negative outcomes. There is a distinct urodynamic presentation for each type of female EUS dysfunction. Sphincteric BoNT-A injection provides a good therapeutic outcome for refractory EUS dysfunction. A narrowing bladder neck and a history of catheterization suggest poor therapeutic outcomes.

## 1. Introduction

Voiding dysfunction (VD) is defined as slow and/or incomplete micturition, which is based on slow urine flow rates and/or high post-void residual (PVR) urine [1]. It is a common bothersome problem for women and represents 7.2%~12.8% of female lower urinary tract symptoms in urology clinics [2]. Detrusor contractility and bladder outlet resistance are two fundamental etiologies of female VD. External urethral sphincter (EUS) dysfunction has been reported to be the most common cause of female bladder outlet obstructions [3]. Among females with VD who are refractory to traditional medications, a large proportion has been found to have EUS dysfunction in video-urodynamic studies (vUDS) [4].

EUS dysfunction can be classified into specific types. Detrusor sphincter dyssynergia (DSD) commonly refers to a condition in which the detrusor contraction is synchronous with contraction of the EUS-striated muscle due to a neurological abnormality [5]. Similar involuntary intermittent contractions of EUS-striated muscles during voiding in neurologically normal patients are defined as dysfunctional voiding (DV) [5]. In addition to DSD and DV, poor relaxation of the external urethral sphincter (PRES) is another subtype of EUS dysfunction. It is characterized by non-relaxed surface electromyography (EMG) activity with narrowing of the distal urethra in the voiding phase of vUDS [4].

Botulinum toxin A (BoNT-A) is a neurotoxin that blocks acetylcholine release from presynaptic nerve terminals and results in a chemo-denervation effect on the skeletal muscle [6]. Sphincteric BoNT-A injection directly blocks the acetylcholine release from the pudendal nerve terminals, resulting in sphincter relaxation in patients with EUS dysfunction. It has served as an option for DSD that is refractory to conservative treatment [7,8]. However, data on the therapeutic effects of sphincteric BoNT-A injection among different types of EUS dysfunction are scarce [9,10]. Most of such data has included gender-mixed populations. Since there is a distinct difference in the lower urinary tract anatomy and function between males and females, it may thus be better to evaluate them separately. This study retrospectively reviews the characteristics of different types of female EUS dysfunctions and their response to sphincteric BoNT-A injections. Additional effort is made to determine the predictive factors related to treatment outcomes.

## 2. Results

A total of 106 females received sphincteric BoNT-A injections during the study period. The mean age at injection was 61.8 ± 19.7 years old; 56 of them had EUS dysfunction without concomitant neurogenic underlying disease, while the others had either central or peripheral neurogenic disorders. Table 1 shows the clinical parameters for female EUS dysfunction before receiving sphincteric BoNT-A injections. Common urodynamic acronyms were listed in the Appendix A Table A1. In patients with idiopathic EUS dysfunction, 23 and 33 patients were diagnosed as DV and PRES, respectively. Patients with DV had significantly higher percentages of DO (83% vs. 0%, *p* < 0.001), higher Pdet (56.3 mL vs. 13.7 cm H_2_O, *p* < 0.001) and BOOI (44.8 vs. 2.1 mL/s, *p* < 0.001), but lower percentages of detrusor underactivity (4% vs. 61%, *p* < 0.001), a lower volume of FS (118.4 vs. 244.8 mL, *p* = 0.016) and US (218.3 vs. 283.5 mL, *p* = 0.032) as compared to patients with PRES.

Among patients with neurogenic EUS dysfunction, 20 were classified as DSD, and 30 were classified as PRES. Similar to subjects with DV, those with DSD had significantly higher percentages of DO (75% vs. 10%, *p* < 0.001), higher Pdet (37.6 mL vs. 12.1 cm H_2_O, *p* < 0.001), BOOI (25.4 vs. 5.5 mL/s, *p* = 0.006) and VE (0.44 vs. 0.19 mL/s, *p* < 0.001), but lower percentages of detrusor underactivity (15% vs. 70%, *p* < 0.001), a lower volume in FSF (106.7 vs. 159.4 mL, *p* = 0.045), US (106.7 vs. 159.4 mL, *p* = 0.045), and PVR (182.2 vs. 296.3 mL, *p* = 0.026) compared to patients with PRES.

Table 2 shows the therapeutic outcomes and urodynamic parameters in female patients with EUS dysfunction after receiving sphincteric BoNT-A injections. The rates of subjective and objective good outcomes ranged between 50 to 75% for each type of EUS dysfunction without obvious statistical differences. In patient with idiopathic EUS dysfunction, both DV and PRES showed improved Qmax (DV: 7.3 to 10.2 mL/s, *p* = 0.004; PRES: 7.4 to 10.2 mL/s, *p* = 0.030) and VE (DV: 0.47 to 0.59 mL/s, *p* = 0.006; PRES: 0.43 to 0.60 mL/s, *p* = 0.008) after the injections. Significantly decreased FS (170.0 vs. 245.9 mL, *p* = 0.023), US (189.3 vs. 286.2 mL, *p* = 0.004) and CBC (307.3 vs, 427.1 mL, *p* = 0.044) were noted in the DV group as compared to the PRES group after the injections. In patients with neurogenic EUS dysfunction, improved Qmax (5.4 to 9.1 mL/s, *p* = 0.002), VE (0.31 to 0.52 mL/s, *p* = 0.001) in UFR and VE (0.14 to 0.31 mL/s, *p* = 0.042) in vUDS were noted in the PRES group after the injections. Trends of improvement in the above parameters were also noted in patients with DSD.

Table 3 shows the baseline clinical characteristics for the patients with subjective good and poor outcomes after the sphincteric BoNT-A injections. Those with a history of catheterization to empty their bladder (61% vs. 28%, *p* = 0.001), narrow BN (81% vs. 9%, *p* < 0.001), PVR ≥230 mL (56% vs. 24%, *p* = 0.001) or CBC ≥350 mL (61% vs. 30%, *p* = 0.002) before the injections had significantly higher poor outcome rates. There were no differences among the patients with different types of EUS dysfunction. The baseline clinical characteristics stratified by objective good and poor outcomes after the sphincteric BoNT-A injections were shown in the Appendix A Table A2. Catheterization history and narrow BN also demonstrated trends of poorer outcomes. The multivariate analysis revealed that a history of catheterization (OR: 0.20, *p* = 0.029) and narrow BN (OR: 0.02, *p* < 0.001) were the significant predictors of poor outcomes after sphincteric BoNT-A injections (Table 4).

## 3. Discussion

Among different types of female EUS dysfunction, significant differences in the image and pressure-flow characteristics were found in our study. Patients diagnosed with DV and DSD presented STU under fluoroscopy, with higher Pdet, BOOI and more frequent DO. On the other hand, patients with PRES were absent of STU and had a higher percentage of DU regardless of whether the neurological underlying disease was present. These distinct findings may imply different underlying pathophysiology. As for the therapeutic effect of sphincteric BoNT-A injection, there was no remarkable difference among types of EUS dysfunction, and the average subjective response rate was 67.0%. Nevertheless, patients with pre-operative BN narrowing or previous usage of catheterization for emptying bladder entailed poor subjective response after receiving sphincteric BoNT-A injection. Patients with pre-operative BN narrowing or previous use of catheterization to empty their bladder gave poor subjective responses after receiving a sphincteric BoNT-A injection.

Female EUS dysfunction has been traditionally classified into DSD or DV based on the presence or absence of neurological abnormalities [5]. PRES was first proposed by Kuo in 2000 [11] as a subtype of DV in men. In the case of women, PRES is characterized as non-relaxed surface EMG activity of the EUS combined with a narrow distal urethra during the voiding phase in the vUDS [4]. There is a lack of reports providing comparisons of these EUS dysfunctions. One previous study found DV to have a poor storage function, including frequent DO, lower FSF, US, CBC and higher Pdet comparing to PRES in patients with bladder pain syndrome [12]. Our studies showed that not only the DV but also the DSD had characteristics of bladder outlet obstruction combined with a decreased storage function compared to PRES in these women after stratifying by the presence of an underlying neurological disease.

The pathophysiology of PRES is believed to be distinct from DV and DSD [8] due to the prominent difference in the urodynamic parameters between them. For DV and DSD patients, the bladder outlet obstruction, induced by the dyssynergic EUS activity during detrusor contraction, is responsible for a high-pressure, low-flow pattern with proximal urethral dilatation [13]. In contrast, poorly relaxed EUS activity caused by an incompletely reversed guarding reflex may have a reflex effect inhibiting the contraction of the detrusor muscle [14,15,16], which may explain the low-pressure low-flow pattern without STU in patients diagnosed with PRES. The hypothesis of a EUS-induced inhibitory effect on the micturition reflex [17] in PRES is supported by the finding of an increased recovery rate of detrusor contractility in patients with DU with concomitant PRES after sphincteric BoNT-A injections [18]. Further basic laboratory research is needed to confirm these assumptions.

Urethra BoNT-A injection has served as an option for refractory voiding dysfunction due to EUS malfunction since 1988 [19]. Many of these studies focused on patients with DSD, but only a handful of them discussed DV and PRES patients. The rates of subjective improvement were around 70%–87%, 61%–88%, and 79%–96% for DV, DSD, and PRES after the sphincteric BoNT-A injection [16] among gender-mixed patients, respectively. These reported outcomes were similar to the results in the present study, where the subjective and objective improvement rates were 65%/74%, 75%/50% for DV, DSD, and 65%/68% for PRES, respectively, in female patients. There was no significant difference in subjective (p = 0.699) or objective (p = 0.317) outcome among difference sphincter dysfunction in our results. Few studies have directly compared the response to urethra BoNT-A injections among the various types of EUS dysfunction. Jiang and his colleagues reported no difference in the rate of satisfactory outcomes between neurogenic and non-neurogenic urethral sphincter hyperactivity after a sphincteric BoNT-A injection [8]. Lee and Kuo demonstrated no significant difference in sphincteric BoNT-A effects among DV, DSD and PRES in a gender-mixed sample [10]. With a larger cohort focusing specifically on females, the present study confirmed that the type of EUS dysfunction does not affect the therapeutic effects of sphincteric BoNT-A injections.

Several predictors of the outcomes for sphincteric BoNT-A injections have been explored. The synergy of BN, detrusor contractility and EUS tone were the most commonly reported ones [20,21]. In the present study, BN narrowing and urethra catheterization pre-operatively was found to be the two main negative therapeutic predictors of subjective outcome in the multivariate analysis. Our data, as a previous study [22], have shown some inconsistencies between subjective satisfaction and objective urodynamic improvements under sphincteric BoNT-A injections. However, the subjective response may be more important than objective results for the patients from their own perspectives. Therefore, we lay emphasis on analyzing subjective outcomes rather than objective ones. A narrowing BN might imply a secondary etiology to the patient’s VD, which leads to a decreased response to a sphincteric BoNT-A injection. Patients who need catheterization pre-operatively may have more complicated etiologies, including poor detrusor contractility, a spastic EUS or psychogenic inhibitory effects that could compromise therapeutic effects. Clinicians should carefully investigate the history and vUDS of patients with refractory VD. With a proper candidate selection and clear explanation before a sphincteric BoNT-A injection, unrealistic expectations could be avoided, and satisfactory outcomes could be achieved.

There are some limitations to this study. First, we did not use validated questionnaires to measure the patient-reported outcomes. However, our subjective response retrieved from medical records may be reliable since it correlated well with the objective urodynamic response and shared similar efficacy to the previously reported data. Second, EUS dysfunction was defined by the image characteristics of vUDS during the voiding phase. A combined needle EMG may be better in terms of assessing EUS activity. However, this procedure would increase the suffering of the patients undergoing the procedure. Third, this was a retrospective study with a relatively small patient number. Nevertheless, we provide a considerable number of patients focusing specifically on female VD compared to recently published data. We also attempted to correct for potential bias by adjusting the significant variables in the statistics. A large prospective study with comprehensive data collection is required for further evaluation of the efficacy of the use of sphincteric BoNT-A injections in different types of female EUS dysfunction.

## 4. Conclusions

Compared to DV and DSD, PRES has distinct urodynamic characteristics that may involve different pathophysiology. Sphincteric BoNT-A injections provide a 67% subjectively good response in general, with similar efficacy among different types of female EUS dysfunction. Narrowing BN and urethra catheterization history were predictors of poor treatment outcomes.

## 5. Materials and Methods

This study was commenced after approval of the Institutional Review Board of the Buddhist Tzu Chi General Hospital, a tertiary medical center in east Taiwan. (IRB 105-151-B). From April 2002 to February 2019, all women who had received urethral sphincteric BoNT-A injections due to refractory VD caused by EUS dysfunction were retrospectively reviewed. EUS dysfunction was diagnosed using vUDS performed in accordance with the International Continence Society (ICS) recommendations [23]. Patients with complicated clinical conditions such as a history of lower urinary tract reconstruction or urethra stenosis were excluded.

Baseline urinary function assessments of each patient were derived from the uroflowmetry (UFR), PVR and vUDS of each patient before the urethra BoNT-A injection. The UFR parameters included voiding volume (VV), maximal flow rate (Qmax), cystometric bladder capacity (CBC), and voiding efficiency (VE). CBC was calculated as the VV plus the PVR. VE was defined as the VV divided by the CBC. The vUDS parameters included the first sensation of filling (FSF), full sensation (FS), urge sensation (US), compliance in the storage phase, the detrusor pressure at the maximal flow rate (Pdet), Qmax, the bladder outlet obstruction index (BOOI), and the PVR, CBC and VE in the voiding phase. The BOOI was calculated as Pdet—2 × Qmax. Major comorbidities (including hypertension, diabetes mellitus, chronic kidney disease, chronic obstructive lung disease and coronary artery disease), a history of a neurogenic disease, a history of catheterization (including Foley, clean intermittent catheterization or suprapubic indwelling) to empty the bladder were collected from the subjects’ medical records.

The types of female VD were determined using vUDS images and the history of the neurogenic disease. DV was defined as contrast stasis at the level of the EUS and presenting with a typical “spinning top” urethra (STU) feature [13] during the voiding phase of the vUDS under conditions without underlying neurological disease. Similar image characteristics combined with a positive neurological underlying disease were defined as DSD. Poor relaxation of the external sphincter (PRES) was defined as narrowing of the distal urethra without the STU feature combined with a non-relaxed surface EMG activity in the voiding phase of the vUDS [4]. The bladder neck (BN) was characterized as narrow if it did not open to a funnel shape during the vUDS voiding phase.

All patients received 100 units of BoNT-A (Allergan, Irvine, CA, USA), which is the most frequent dosage in contemporary publications [16], injected in the EUS after the clinical evaluation. The BoNT-A (100 U) was diluted in 5 mL of normal saline, which created a total of five injections with 27-gauge 1 mL syringe needles. The injections were performed circumferentially (except at the 6 o’clock position) into the urethral sphincter via a perineal route in the operating room under general anesthesia. Detailed techniques used in a urethral injection were described in a previous series [24]. The subjective outcomes were graded as “good” or “poor” according to the patient’s perceptions of VD improvement as expressed in the medical records. Objective outcomes were graded as “good” if there was a 50% improvement in the Qmax or PVR after the injection. Both subjective and objective outcomes were assessed 1 month after the injections. Baseline and post-treatment urodynamic parameters were further analyzed for the treatment effects of the sphincteric BoNT-A injection.

Continuous and categorical variables were expressed as the mean ± standard deviation and number (percentage), respectively. Between-group differences were examined with an independent t-test on the continuous variables and a Chi-square test on the categorical variables. Fisher’s Exact Test was applied if more than 20% of the expected frequencies were less than five. The changes in the variables after treatment compared to the within-group baseline were tested separately with a paired sample t-test and the McNemar test for the continuous and categorical variables. Between-group differences in the variables after treatment adjusted under the pre-treatment condition were evaluated with an analysis of covariance (ANCOVA) and a univariate logistic regression for the continuous and categorical variables, respectively. Variables demonstrating significant differences between subjective good and poor outcomes were further analyzed with a multivariate logistic regression to discern the predictors of the subjective outcomes. All analyses were performed through SPSS Statistics for Windows, Version 17.0. Chicago: SPSS Inc. Two-sided *p*-values less than 0.05 were considered significant.

## Figures and Tables

**Table 1:** Basic clinical parameters for female patients with sphincter dysfunction before receiving sphincteric botulinum toxin A injections.

	Idiopathic Sphincter Dysfunction (N = 56)					Neurogenic Sphincter Dysfunction (N = 50)				
	DV (N = 23)		PRES (N = 33)		*p*	DSD (N = 20)		PRES (N = 30)		*p*
HT	4	(17%)	17	(52%)	0.012 *	8	(40%)	12	(40%)	1.000
DM	4	(17%)	8	(24%)	0.743	9	(45%)	11	(37%)	0.556
CKD	0	(0%)	2	(6%)	0.507	1	(5%)	3	(10%)	0.641
COPD	1	(4%)	0	(0%)	0.411	2	(10%)	0	(0%)	0.155
CAD	0	(0%)	4	(12%)	0.136	2	(10%)	1	(3%)	0.556
Cathterization ^a^	12	(52%)	9	(27%)	0.058	8	(40%)	13	(43%)	0.815
Baseline UFR Parameters										
VV	139.7	±94.3	130.5	±125.6	0.770	107.9	±68.5	109.4	±85.3	0.946
Qmax	7.3	±3.2	7.4	±5.4	0.941	7.6	±5.9	5.4	±3.7	0.156
PVR	160.8	±98.5	188.1	±147.5	0.443	208.9	±196.3	258.5	±151.1	0.318
CBC	300.4	±135.4	318.7	±127.8	0.617	316.7	±195.3	367.9	±167.2	0.326
VE	0.47	±0.23	0.43	±0.32	0.611	0.44	±0.26	0.31	±0.22	0.067
Baseline vUDS Parameters										
DO	19	(83%)	0	(0%)	<0.001 *	15	(75%)	3	(10%)	<0.001 *
DU	1	(4%)	20	(61%)	<0.001 *	3	(15%)	21	(70%)	<0.001 *
DHIC	2	(9%)	6	(18%)	0.449	0	(0%)	6	(20%)	0.069
Narrow BN	6	(26%)	12	(36%)	0.418	4	(20%)	12	(40%)	0.137
FSF	117.8	±82.5	154.7	±67.6	0.072	106.7	±52.5	159.4	±106.0	0.045 *
FS	184.4	±86.0	244.8	±92.0	0.016 *	166.8	±101.8	217.7	±107.5	0.101
US	218.3	±108.2	283.5	±109.2	0.032 *	183.0	±114.2	249.5	±110.0	0.045 *
CBC	335.5	±119.0	390.6	±208.0	0.257	280.0	±202.0	365.8	±157.1	0.098
Compliance	82.8	±113.1	81.3	±84.1	0.953	59.8	±118.6	62.3	±109.6	0.940

BN: bladder neck, BOOI: bladder outlet obstruction index, CAD: coronary artery disease, CBC: cystometric bladder capacity, CKD: chronic kidney disease, COPD: chronic obstructive lung disease, DM: diabetes mellitus, DO: detrusor overactivity, DHIC: detrusor hyperactivity with impaired contractility, DSD: detrusor sphincter dyssynergia, DU: detrusor underactivity, DV: dysfunctional voiding, HT: hypertension, FS: full sensation, FSF: the first sensation of filling, Pdet: detrusor pressure at a maximum flow rate, PRES: poor relaxation of the external sphincter, PVR: post-void residual volume, Qmax: maximal flow rate, UFR: uroflowmetry, US: urge sensation, VE: voiding efficiency, vUDS: video-urodynamic studies, VV: voiding volume. ^a^ Catheterization included clean intermittent catheterization, Foley or cystostomy. * *p* < 0.05.

**Table 2:** Therapeutic outcomes and urodynamic parameters in female patients with sphincter dysfunction receiving sphincteric botulinum toxin A injections.

		Idiopathic Voiding Dysfunction (N = 56)				Neurogenic Voiding Dysfunction (N = 50)					
		DV (N = 23)		PRES (N = 33)		*p* ^a^	DSD (N = 20)		PRES (N = 30)		*p* ^a^
Good sub. Outcome		15	(65%)	21	(64%)	0.903	15	(75%)	20	(67%)	0.529
Good obj. Outcome		17	(74%)	22	(67%)	0.562	10	(50%)	21	(70%)	0.220
UFR Parameters											
Qmax	B	7.3	±3.2	7.4	±5.4	0.980	8.3	±5.9	5.4	±3.7	0.800
	P	10.2 **	±5.7	10.2 *	±7.7		10.8	±8.6	9.1 **	±7.2	
VV	B	139.7	±94.3	130.5	±125.6	0.559	109.7	±69.9	109.4	±85.3	0.921
	P	174.7	±104.7	190.9	±124.6		125.1	±73.7	122.8	±80.7	
PVR	B	160.8	±98.5	188.1	±147.5	0.657	209.7	±201.6	258.5	±151.1	0.802
	P	134.1	±99.4	171.0	±221.8		144.5	±142.3	176.9	±191.6	
CBC	B	300.4	±135.4	318.7	±127.8	0.328	319.4	±200.3	367.9	±167.2	0.831
	P	308.8	±118.9	360.4	±203.5		269.6	±161.7	299.6	±188.1	
VE	B	0.47	±0.23	0.43	±0.32	0.616	0.45	±0.26	0.31	±0.22	0.579
	P	0.59 **	±0.30	0.60 **	±0.32		0.53	±0.27	0.52 **	±0.31	
vUDS Parameters											
FSF	B	117.7	±89.6	164.4	±74.3	0.086	109.4	±49.7	142.9	±61.0	0.710
	P	110.4	±69.8	151.8	± 72.4		117.5	±56.7	134.2	±48.5	
FS	B	191.7	±90.9	250.3	± 108.3	0.023	180.4	±116.5	200.7	±68.5	0.700
	P	163.3	±77.4	251.7	± 109.8		170.8	±56.6	183.0	±75.7	
US	B	228.9	±111.9	286.3	± 127.0	0.004	195.3	±129.4	237.8	±80.3	0.600
	P	180.3	±83.4	293.8	±114.1		187.4	±65.6	208.6	±92.1	
Compliance	B	92.4	±125.4	74.4	±87.5	0.194	75.8	±151.6	49.3	±66.9	0.540
	P	44.5	±35.6	65.5	±66.4		35.6	±21.9	29.0	±19.6	
Pdet	B	53.6	±29.0	13.0	±11.9	0.136	42.1	±23.5	8.3	±11.6	0.635
	P	51.1	±31.2	11.9	±15.4		31.2	±15.4	17.3	±19.1	
Qmax	B	5.8	±2.5	4.2	±5.4	0.334	6.3	±5.4	2.6	±3.5	0.809
	P	6.3	±4.3	6.7	±6.6		7.5	±10.3	5.1	±6.1	
BOOI	B	41.9	±30.8	4.6	±11.3	0.116	29.4	±29.8	3.2	±13.5	0.421
	P	38.5	±33.0	-1.5	±15.9		16.2	±19.8	7.2	±24.8	
PVR	B	217.2	±128.2	314.3	±248.6	0.118	249.2	±235.5	306.2	±150.3	0.970
	P	174.4	±135.9	305.2	±263.1		248.3	±209.4	275.7	±196.8	
CBC	B	340.5	±126.7	402.8	±237.1	0.044	347.8	±222.3	366.4	±165.7	0.264
	P	303.9	±141.6	430.0	±198.3		323.1	±183.9	393.0	±169.8	
VE	B	0.40	±0.22	0.22	±0.27	0.769	0.34	±0.26	0.14	±0.18	0.676
	P	0.47	±0.33	0.39	±0.38		0.35	±0.37	0.31 *	±0.37	

B: before injection, BOOI: bladder outlet obstruction index, CBC: cystometric bladder capacity, DSD: detrusor sphincter dyssynergia, DV: dysfunctional voiding, FS: full sensation, FSF: the first sensation of filling, obj.: objective, P: post-injection, Pdet: detrusor pressure at maximum flow rate, PRES: poor relaxation of the external sphincter, PVR: post-void residual volume, Qmax: the maximal flow rate, sub.: subjective, UFR: uroflowmetry, US: urge sensation, VE: voiding efficiency, vUDS: video-urodynamic studies, VV: voiding volume. ^a^ Between-group differences after treatment adjusting by the pre-treatment condition. * Within-group differences in changes after treatment < 0.05. ** Within-group differences in changes after treatment < 0.01.

**Table 3:** Baseline clinical characteristics stratified by subjective outcomes.

	Good Outcome (N = 71)	Poor Outcome (N = 35)	*p*
Age	61.5 ± 18.9	62.4 ± 21.3	0.814
HT	28 (39%)	14 (39%)	0.956
DM	24 (34%)	8 (22%)	0.216
CKD	4 (6%)	2 (6%)	1.000
COPD	2 (3%)	1 (3%)	1.000
CAD	5 (7%)	2 (6%)	1.000
Neurogenic history ^a^	35 (49%)	15 (42%)	0.455
Catheterization ^b^	20 (28%)	22 (61%)	0.001 *
DV	15 (21%)	8 (23%)	0.839
DSD	15 (21%)	5 (14%)	0.397
PRES	41 (58%)	22 (63%)	0.614
DO	26 (37%)	12 (33%)	0.737
DU	29 (41%)	16 (44%)	0.722
DHIC	9 (13%)	5 (14%)	1.000
Narrow BN	6 (9%)	29 (81%)	<0.001 *
**UFR parameters**			
Qmax	7.3 ± 4.7	5.8 ± 4.4	0.096
Voiding volume	129.5 ± 103.7	109.5 ± 85.3	0.327
PVR (≥230 mL)	17 (24%)	20 (56%)	0.001 *
CBC (≥350 mL)	21 (30%)	22 (61%)	0.002 *
VE	0.44 ± 0.26	0.35 ± 0.27	0.089
**vUDS parameters**			
FSF	148.4 ± 92.2	118.7 ± 56.7	0.080
FS	218.7 ± 107.0	187.6 ± 84.0	0.130
US	251.4 ± 120.9	215.8 ± 99.4	0.130
Compliance	78.6 ± 116.3	58.1 ± 72.0	0.335
Pdet	28.9 ± 29.6	22.8 ± 24.0	0.285
Qmax	5.7 ± 5.1	4.2 ± 3.7	0.095
BOOI	17.6 ± 30.7	14.4 ± 25.6	0.567
PVR	226.8 ± 178.5	279.4 ± 190.2	0.162
CBC	339.9 ± 172.1	372.2 ± 189.3	0.376
VE	0.35 ± 0.30	0.27 ± 0.22	0.090

BN: bladder neck, BOOI: bladder outlet obstruction index, CAD: coronary artery disease, CBC: cystometric bladder capacity, CKD: chronic kidney disease, COPD: chronic obstructive lung disease, DM: diabetes mellitus, DO: detrusor overactivity, DHIC: detrusor hyperactivity with impaired contractility, DSD: detrusor sphincter dyssynergia, DU: detrusor underactivity, DV: dysfunctional voiding, HT: hypertension, FS: full sensation, FSF: the first sensation of filling, Pdet: detrusor pressure at the maximum flow rate, PRES: poor relaxation of the external sphincter, PVR: post-void residual volume, Qmax: the maximal flow rate, UFR: uroflowmetry, US: urge sensation, VE: voiding efficiency, vUDS: video-urodynamic studies. ^a^ Diabetes was included in neurogenic history. ^b^ Catheterization included clean intermittent catheterization, Foley, or cystostomy. * *p* < 0.05.

**Table 4:** Logistic regression for predictors of good subjective outcomes before sphincteric botulinum toxin A injection.

	Univariate Analysis				Multivariate Analysis			
	OR	95% CI	95% CI	*p*	OR	95% CI	95% CI	*p*
Age	0.99	0.98	1.02	0.812				
Neurogenic history ^a^	1.361	0.61	3.06	0.455				
Catheterization ^b^	0.25	0.11	0.58	0.001 *	0.20	0.05	0.85	0.029 *
DV	0.90	0.34	2.39	0.839				
DSD	1.61	0.53	4.85	0.400				
PRES	0.81	0.35	1.86	0.615				
Narrow BN	0.02	0.01	0.07	<0.001 *	0.02	0.00	0.07	<0.001 *
PVR (≥230 mL)	0.25	0.11	0.59	0.002 *	0.33	0.05	2.24	0.254
CBC (≥350 mL)	0.27	0.12	0.62	0.002 *	0.88	0.15	5.33	0.888

BN: bladder neck, CBC: cystometric bladder capacity, DSD: detrusor sphincter dyssynergia, DV: dysfunctional voiding, PRES: poor relaxation of the external sphincter, PVR: post-void residual volume. ^a^ Diabetes was included in neurogenic history. ^b^ Catheterization included clean intermittent catheterization, Foley, or cystostomy. * *p* < 0.05.

## Data Availability

Not applicable.

## Appendix A

**Table A1:** Acronyms of common urodynamic parameters.

BN	Bladder neck	BOOI	Bladder outlet obstruction index
CBC	Cystometric bladder capacity	DHIC	Detrusor hyperactivity with impaired contractility
DO	Detrusor over-activity	DSD	Detrusor sphincter dyssynergia
DU	Detrusor under-activity	DV	Dysfunctional voiding
FS	Full sensation	FSF	The first sensation of filling
Pdet	Detrusor pressure at the maximum flow rate	PRES	Poor relaxation of the external sphincter
PVR	Post-void residual volume	Qmax	Maximal flow rate
UFR	Uroflowmetry	US	Urge sensation
VE	Voiding efficiency	vUDS	Video-urodynamic studies
VV	Voiding volume		

**Table A2:** Baseline clinical characteristics stratified by objective outcomes.

	Good Outcome (N = 71)	Poor Outcome (N = 35)	*p*
Age	62.6 ± 18.8	60.2 ± 21.8	0.553
HT	30 (42%)	11 (31%)	0.282
DM	23 (32%)	8 (23%)	0.310
CKD	3 (4%)	3 (9%)	0.362
COPD	0 (0%)	3 (9%)	0.034 *
CAD	6 (9%)	1 (3%)	0.421
Neurogenic history ^a^	31 (44%)	18 (51%)	0.451
Catheterization ^b^	24 (34%)	18 (51%)	0.081
DV	17 (24%)	6 (17%)	0.404
DSD	10 (14%)	9 (26%)	0.152
PRES	43 (61%)	20 (57%)	0.673
DO	27 (38%)	11 (31%)	0.505
DU	30 (42%)	15 (43%)	0.953
DHIC	11 (16%)	3 (9%)	0.379
Narrow BN	19 (27%)	16 (46%)	0.051
**UFR parameters**			
Qmax	6.5 ± 4.6	7.6 ± 4.8	0.241
Voiding volume	112.3 ± 94.7	145.5 ± 103.2	0.104
PVR (≥230 mL)	27 (38%)	10 (29%)	0.337
CBC (≥350 mL)	29 (41%)	14 (40%)	0.934
VE	0.36 ± 0.25	0.50 ± 0.28	0.015 *
**vUDS parameters**			
FSF	145.8 ± 93.3	123.8 ± 56.3	0.202
FS	218.9 ± 109.2	188.1 ± 78.9	0.139
US	254.3 ± 123.1	209.5 ± 92.5	0.060
Compliance	79.0 ± 116.1	58.6 ± 73.1	0.345
Pdet	28.5 ± 29.2	24.1 ± 25.4	0.445
Qmax	5.4 ± 4.6	4.8 ± 5.0	0.560
BOOI	17.7 ± 29.7	14.5 ± 28.0	0.588
PVR	276.1 ± 196.0	181.7 ± 139.7	0.005 *
CBC	379.8 ± 172.0	294.4 ± 180.1	0.020 *
VE	0.32 ± 0.28	0.34 ± 0.27	0.679

BN: bladder neck, BOOI: bladder outlet obstruction index, CAD: coronary artery disease, CBC: cystometric bladder capacity, CKD: chronic kidney disease, COPD: chronic obstructive lung disease, DM: diabetes mellitus, DO: detrusor overactivity, DHIC: detrusor hyperactivity with impaired contractility, DSD: detrusor sphincter dyssynergia, DU: detrusor underactivity, DV: dysfunctional voiding, HT: hypertension, FS: full sensation, FSF: the first sensation of filling, Pdet: detrusor pressure at the maximum flow rate, PRES: poor relaxation of the external sphincter, PVR: post-void residual volume, Qmax: maximal flow rate, UFR: uroflowmetry, US: urge sensation, VE: voiding efficiency, vUDS: video-urodynamic studies. ^a^ Diabetes was included in neurogenic history. ^b^ Catheterization included clean intermittent catheterization, Foley, or cystostomy. * *p* < 0.05.
